# Clinical and autonomic correlates of chronic Sialorrhea in Parkinson's disease: a questionnaire-based study with cluster analysis of autonomic and dysphagia burden

**DOI:** 10.1016/j.prdoa.2026.100467

**Published:** 2026-06-18

**Authors:** Shohei Okusa, Tomonori Nukariya, Yoshihiro Nihei, Jin Nakahara, Morinobu Seki

**Affiliations:** a Department of Neurology, Keio University School of Medicine, Tokyo, Japan; b Parkinson's Disease Center, Keio University Hospital, Tokyo, Japan; c Center for Parkinson's disease Research, Keio University, Tokyo, Japan

**Keywords:** Parkinson's disease, Chronic sialorrhea, Dysphagia, Autonomic dysfunction, Botulinum toxin therapy

## Abstract

**Introduction:** Chronic sialorrhea is common in Parkinson's disease (PD), but its clinical correlates and patient awareness of treatment options remain unclear. We examined factors associated with drooling, focusing on autonomic dysfunction and dysphagia, and assessed perceptions of botulinum toxin therapy.

**Methods:** We conducted a questionnaire-based study of consecutive PD outpatients at Keio University Hospital from October to December 2025. Drooling was defined using MDS-UPDRS Part II item 2. Autonomic symptoms were assessed with SCOPA-AUT and swallowing with the Swallowing Disturbances Questionnaire (SDQ). Clinical variables and ^123^I-metaiodobenzylguanidine myocardial scintigraphy indices were extracted from medical records. Multivariable logistic regression identified factors independently associated with drooling. K-means clustering (k = 3) was also performed among droolers using standardized SCOPA-AUT and SDQ scores.

**Results:** Among 208 patients, 116 (55.8%) were classified as droolers. Droolers were more often male and had longer disease and treatment duration, higher Hoehn and Yahr stage, higher levodopa equivalent daily dose, higher SDQ scores, more severe autonomic symptoms, and lower MIBG heart-to-mediastinum ratios. In multivariable analysis, higher SCOPA-AUT and SDQ scores remained independently associated with drooling. Only 34.5% of droolers perceived drooling as problematic, 14.7% were aware of botulinum toxin therapy, and 21.6% were willing to receive injections. Clustering identified three phenotypes: low autonomic/low dysphagia, high autonomic/high dysphagia, and high autonomic/low dysphagia.

**Conclusion:** Chronic sialorrhea in PD is associated with dysphagia burden and broader autonomic dysfunction, suggesting heterogeneous mechanisms. Limited awareness highlights the need for systematic screening and patient education.

## Highlights

• Drooling affected 55.8% of PD outpatients in a questionnaire-based study.
• Droolers had higher dysphagia (SDQ) and broader autonomic burden (SCOPA-AUT).
• Autonomic differences persisted after excluding the drooling-related SCOPA-AUT item.
• In droolers, 34.5% perceived drooling as problematic; 21.6% accepted injections.
• Clustering analysis identified 3 phenotypes by autonomic and dysphagia severity.

## 1 Introduction

Chronic sialorrhea is a condition in which the volume of saliva produced exceeds the amount effectively swallowed, resulting in spillage from the oral cavity. It occurs secondary to various neurological and neuromuscular disorders, including Parkinson's disease (PD), muscular dystrophy, stroke, amyotrophic lateral sclerosis, cerebral palsy, and traumatic brain injury. Sialorrhea can cause substantial physical morbidity, such as perioral skin maceration and breakdown, deterioration of oral hygiene, dysphagia, dysarthria, sleep disturbance, and aspiration pneumonia. [1], [2] In addition, it imposes significant psychosocial burdens, including embarrassment and stigma, social withdrawal due to avoidance of physical contact with others, and increased caregiver burden related to frequent clothing changes and laundry. [3] Despite its negative impact on quality of life (QOL) for both patients and caregivers, sialorrhea is often underreported by patients and clinical attention from healthcare professionals has been insufficient. [4], [5], [6] One contributing factor is that effective, targeted treatment options have historically been limited, with management largely restricted to symptomatic approaches such as exploiting anticholinergic side effects or implementing rehabilitation-based interventions. [7], [8], [9]

In recent years, botulinum toxin therapy has emerged as an established treatment option for chronic sialorrhea. [10], [11] In Japan, incobotulinumtoxin A received an additional indication for chronic sialorrhea in June 2025, marking a significant shift in the clinical management of this symptom. Notably, patients with PD constituted the majority of participants in a domestic phase III clinical trial, [11] underscoring the clinical relevance of sialorrhea in this population. Indeed, chronic sialorrhea is common in PD, with reported prevalence rates of up to 84%. [12] Several studies have suggested that salivary production in PD is not increased and may even be reduced compared with that in age-matched controls, indicating that sialorrhea in PD is more likely attributable to impaired saliva clearance rather than hypersalivation. [13], [14] Impaired clearance may result from dysphagia, reduced spontaneous swallowing frequency, and postural factors such as stooped posture. [15] Although sialorrhea is frequently associated with dysphagia, severe dysphagia does not necessarily precede sialorrhea, and the two symptoms are considered at least partially independent. [16] Moreover, sialorrhea has been linked to non-motor autonomic dysfunction, and patients who experience drooling often exhibit more severe and widespread autonomic impairment. [6], [17] However, the extent to which autonomic dysfunction independently contributes to drooling, beyond disease severity and dysphagia, remains insufficiently understood. Several studies have also reported a higher prevalence of sialorrhea in male patients with PD, although the mechanisms underlying this sex difference remain incompletely understood. [15], [17]

Against this background and in the context of the recent approval of botulinum toxin therapy for chronic sialorrhea in Japan, there is a growing need to better characterize the clinical profiles of patients with PD who experience drooling. We therefore conducted a questionnaire survey of consecutive PD outpatients at our institution. The aims of this study were to identify clinical and autonomic factors associated with chronic sialorrhea in Japanese patients with PD, assess patient awareness and perceptions of newly available treatment options, and clarify the relative contributions of motor symptoms, autonomic dysfunction, and dysphagia to chronic sialorrhea.

## 2 Subjects and methods

### 2.1 Study design and subjects

This observational study represents a retrospective analysis of prospectively collected questionnaire and clinical data from consecutive patients with PD who attended the outpatient clinic at Keio University Hospital between October 23 and December 25, 2025. A total of 212 patients agreed to participate. All patients were diagnosed with clinically established or probable PD based on the Movement Disorder Society (MDS) clinical diagnostic criteria. [18]

This study was approved by the institutional ethics committee of Keio University School of Medicine (approval number: 20231166). The requirement for written informed consent was waived owing to the retrospective nature of the analysis, and information about the study was disclosed to all patients, who were given the opportunity to opt out.

### 2.2 Clinical data and questionnaires

Demographic and clinical data, including age, sex, age at onset, disease duration, treatment duration, Hoehn & Yahr (HY) stage, medication profiles, past history, and results of ^123^I-metaiodobenzylguanidine (MIBG) myocardial scintigraphy, were extracted from medical records.

In addition, patients completed a self-report questionnaire that we developed for this study, which assessed subjective worsening of drooling and perceptions of botulinum toxin therapy. The severity of drooling was assessed using item 2 of the MDS–Unified Parkinson's Disease Rating Scale (MDS-UPDRS) Part II. [19] Patients who responded “normal” were categorized as non-droolers, whereas those selecting “very mild” to “severe” were classified as droolers. Autonomic symptoms were assessed using the Japanese version of the Scales for Outcomes in Parkinson's Disease–Autonomic (SCOPA-AUT). [20] The SCOPA-AUT consists of 25 items across seven domains: gastrointestinal (items 1–7), urinary (items 8–13), cardiovascular (items 14–16), thermoregulatory (items 17, 18, 20, and 21), pupillomotor (item 19), sexual function in men (items 22 and 23), and sexual function in women (items 24 and 25). Swallowing function was assessed using the Japanese version of the Swallowing Disturbances Questionnaire (SDQ). [21] Among patients classified as droolers, additional assessments included the Drooling Severity and Frequency Scale (DSFS), item 3 of the MDS-UPDRS Part II, and non-motor symptom measures related to drooling and fluctuations, including items J-1 and J-2 and the non-motor fluctuation severity (0–4, not present-severe) of the MDS–Non-Motor Symptoms Scale (MDS-NMS). [19], [22], [23]

### 2.3 Statistical analysis

Demographic and clinical data are presented as frequencies or means ± standard deviation, as appropriate. Between-group comparisons were performed using the Mann–Whitney *U* test for continuous variables. Comparisons among three groups were conducted using the Kruskal–Wallis test with Bonferroni correction for post hoc analyses. Effect sizes for continuous variables were calculated using Cohen's d. Multivariable logistic regression analysis was conducted to identify factors independently associated with drooling. The presence of drooling (MDS-UPDRS Part II item 2 ≥ 1) was included as the dependent variable. SCOPA-AUT score, SDQ score, and MIBG heart-to-mediastinum (H/M) ratio were entered as primary explanatory variables. Age, sex, disease duration, HY stage, and levodopa equivalent daily dose (LEDD) were included in the model as potential confounders. All variables were simultaneously entered into the model using the forced entry method. An unsupervised clustering analysis was performed among patients classified as droolers using two continuous variables: the SCOPA-AUT total score and the SDQ score. Prior to clustering, both variables were standardized to z-scores (mean = 0, standard deviation = 1) to account for differences in scale and variance. Clusters were identified using the k-means algorithm with Euclidean distance. We explored 2 to 5 cluster solutions and selected the optimal number of clusters based on a combination of the elbow method, silhouette coefficients, and clinical interpretability. The final model used k = 3 clusters to stratify patients into clinically interpretable profiles based on autonomic symptom burden and dysphagia severity. After k-means clustering, each participant was assigned to the cluster corresponding to the nearest centroid. Cluster centroids were back-transformed to the original scale for interpretability. Cluster membership was summarized by group size and descriptive statistics, and visualized using a two-dimensional scatter plot with cluster membership indicated by color and cluster centroids marked. Statistical analyses were performed in SPSS software (version 29.0; IBM Corp., Armonk, NY, USA). Clustering was performed in Python using scikit-learn (StandardScaler and KMeans), with matplotlib for visualization. A two-tailed *p*-value <0.05 was considered statistically significant.

## 3 Results

### 3.1 Clinical and autonomic profiles of droolers and non-droolers (Table 1)

Four patients with PD had incomplete questionnaire responses and were excluded; thus, 208 patients were included in the final analysis. Of these, 116 (55.8%) were classified as droolers and 92 (44.2%) as non-droolers. Droolers were more frequently male than non-droolers (63.8% vs 45.7%, *p* = 0.009). Age and age at onset did not differ significantly between the two groups. Droolers had a longer disease duration (9.6 ± 6.4 vs 7.4 ± 5.8 years, *p* = 0.007) and longer treatment duration (6.9 ± 5.8 vs 4.7 ± 5.5 years, *p* < 0.001). Disease severity was greater in droolers, with higher HY stage (2.9 ± 0.8 vs 2.5 ± 0.8, *p* = 0.001) and higher LEDD (803.0 ± 516.9 vs 603.2 ± 448.0, *p* < 0.001). The use of anticholinergic medication and the prevalence of diabetes mellitus or heart disease did not differ between groups. On cardiac sympathetic imaging, droolers exhibited lower MIBG H/M ratios than non-droolers in both early (1.86 ± 0.53 vs 2.01 ± 0.52, *p* = 0.013) and delayed images (1.67 ± 0.63 vs 1.78 ± 0.55, *p* = 0.044), whereas washout rates did not differ significantly (*p* = 0.21). Autonomic dysfunction was more pronounced in droolers, with higher SCOPA-AUT total scores (16.8 ± 7.6 vs 10.5 ± 6.4, *p* < 0.001). This difference remained significant even after excluding the drooling-related SCOPA-AUT item (15.3 ± 7.3 vs 10.4 ± 6.3, *p* < 0.001). Domain-level analyses showed higher gastrointestinal (6.2 ± 3.2 vs 3.1 ± 2.8, *p* < 0.001), urinary (6.6 ± 3.9 vs 4.4 ± 2.9, *p* < 0.001), and cardiovascular subdomains (1.2 ± 1.3 vs 0.8 ± 1.1, *p* = 0.011), whereas thermoregulatory, pupillomotor, and sexual function subscores were not significantly different. Dysphagia burden was also greater in droolers, as reflected by higher SDQ scores (7.1 ± 5.5 vs 2.7 ± 3.0, *p* < 0.001).

**Table 1:** Clinical and autonomic profiles of droolers vs non-droolers with Parkinson's disease.

	Total patients	Non-droolers	Droolers	*p* value
N	208	92 (44.2)	116 (55.8)	–
Male	116 (55.8)	42 (45.7)	74 (63.8)	0.009
Age, yrs	70.8 ± 10.2	69.5 ± 10.7	71.9 ± 9.7	0.06
Onset age, yrs	62.2 ± 12.1	62.1 ± 12.5	62.3 ± 11.8	0.96
Disease duration, yrs	8.6 ± 6.2	7.4 ± 5.8	9.6 ± 6.4	0.007
Treatment duration, yrs	5.9 ± 5.8	4.7 ± 5.5	6.9 ± 5.8	<0.001
Hoehn & Yahr stage	2.7 ± 0.8	2.5 ± 0.8	2.9 ± 0.8	0.001
LEDD	714.6 ± 496.5	603.2 ± 448.0	803.0 ± 516.9	<0.001
Use of anticholinergic medication	19 (9.1)	10 (10.9)	9 (7.8)	0.46
History of diabetes	12 (5.8)	4 (4.4)	8 (6.9)	0.43
History of heart disease	18 (8.7)	7 (7.6)	11 (9.5)	0.63
MIBG myocardial scintigraphy				
H/M ratio early image (*n* = 179)	1.92 ± 0.53	2.01 ± 0.52	1.86 ± 0.53	0.013
H/M ratio delayed image (*n* = 173)	1.72 ± 0.60	1.78 ± 0.55	1.67 ± 0.63	0.044
Washout rate (*n* = 170)	52.5 ± 17.1	50.7 ± 16.9	54.0 ± 17.2	0.21
MDS-UPDRS Part II item 2	1.3 ± 1.4	0	2.4 ± 1.0	–
SCOPA-AUT total score	14.0 ± 7.7	10.5 ± 6.4	16.8 ± 7.6	<0.001
SCOPA-AUT total score excluding the drooling item	13.1 ± 7.3	10.4 ± 6.3	15.3 ± 7.3	<0.001
Gastrointestinal	4.8 ± 3.4	3.1 ± 2.8	6.2 ± 3.2	<0.001
Urinary	5.6 ± 3.6	4.4 ± 2.9	6.6 ± 3.9	<0.001
Cardiovascular	1.0 ± 1.3	0.8 ± 1.1	1.2 ± 1.3	0.011
Thermoregulatory	1.5 ± 2.0	1.3 ± 1.8	1.7 ± 2.1	0.24
Pupillomotor	0.5 ± 0.8	0.5 ± 0.8	0.5 ± 0.8	0.44
Sexual function (male)	0.8 ± 1.6	0.9 ± 1.6	0.8 ± 1.5	0.59
Sexual function (female)	0.2 ± 0.9	0.1 ± 0.5	0.3 ± 1.1	0.31
SDQ score	5.1 ± 5.1	2.7 ± 3.0	7.1 ± 5.5	<0.001

The data are mean ± SD or n (%).

Abbreviations: H/M: heart-to-mediastinum; LEDD: levodopa equivalent daily doses; MDS: Movement Disorder Society; MIBG: 123 I-metaiodobenzylguanidine; SCOPA-AUT: Scales for Outcomes in Parkinson's Disease-Autonomic; SDQ: Swallowing Disturbances Questionnaire (Japanese version); UPDRS: Unified Parkinson's Disease Rating Scale.

In multivariable logistic regression analysis, both SCOPA-AUT total score (excluding the drooling-related item) and SDQ score were independently associated with drooling after adjustment for age, sex, disease duration, Hoehn and Yahr stage, and LEDD (Supplementary Table S1, Model 1). When SCOPA-AUT subdomains were entered into the model, the gastrointestinal domain score excluding the drooling item and urinary domains remained significantly associated with drooling (Supplementary Table S1, Model 2).

### 3.2 Sex-related differences in clinical and autonomic profiles (Table 2)

In the overall cohort (116 males and 92 females), age, age at onset, disease duration, treatment duration, and HY stage did not differ significantly by sex (all *p* > 0.05). Male patients had a higher prevalence of heart disease than females (12.9% vs 3.3%, *p* = 0.014). Males also demonstrated lower early MIBG H/M ratios compared with females (1.84 ± 0.48 vs 2.04 ± 0.57, *p* = 0.010). Male patients showed greater drooling-related disability, as indicated by higher scores on MDS-UPDRS Part II item 2 (1.6 ± 1.5 vs 1.0 ± 1.2, *p* = 0.005), and higher SCOPA-AUT total scores than female patients (15.4 ± 7.6 vs 12.4 ± 7.6, *p* = 0.002). This difference remained significant even after excluding the drooling-related item and the sexual function subdomain. At the domain level, gastrointestinal (5.4 ± 3.5 vs 4.2 ± 3.1, *p* = 0.019), urinary (6.4 ± 3.6 vs 4.7 ± 3.5, *p* < 0.001), and sexual function subscores (0.8 ± 1.6 vs 0.2 ± 0.9, *p* = 0.002) were higher in males. SDQ scores did not differ significantly by sex (*p* = 0.24).

**Table 2:** Comparison of clinical and autonomic profiles by sex among patients with Parkinson's disease.

	Male	Female	*p* value
N	116 (55.8)	92 (44.2)	–
Age, yrs	71.0 ± 9.43	70.58 ± 11.1	1.00
Onset age, yrs	62.9 ± 11.6	61.3 ± 12.6	0.46
Disease duration, yrs	8.1 ± 5.9	9.3 ± 6.5	0.15
Treatment duration, yrs	5.6 ± 5.3	6.4 ± 6.3	0.43
Hoehn & Yahr stage	2.6 ± 0.8	2.9 ± 0.9	0.060
LEDD	746.1 ± 539.1	675.0 ± 436.6	0.49
Use of anticholinergic medication	8 (6.9)	11 (12.0)	0.21
History of diabetes	8 (6.9)	4 (4.4)	0.43
History of heart disease	15 (12.9)	3 (3.3)	0.014
MIBG myocardial scintigraphy			
H/M ratio early image (n = 179)	1.84 ± 0.48	2.04 ± 0.57	0.010
H/M ratio delayed image (n = 173)	1.65 ± 0.56	1.82 ± 0.64	0.061
Washout rate (n = 170)	53.7 ± 18.4	50.8 ± 15.2	0.26
MDS-UPDRS Part II item 2	1.6 ± 1.5	1.0 ± 1.2	0.005
SCOPA-AUT total score	15.4 ± 7.6	12.4 ± 7.6	0.002
SCOPA-AUT total score excluding the drooling and sexual function items	13.4 ± 6.8	11.5 ± 7.1	0.019
Gastrointestinal	5.4 ± 3.5	4.2 ± 3.1	0.019
Urinary	6.4 ± 3.6	4.7 ± 3.5	<0.001
Cardiovascular	1.1 ± 1.3	0.9 ± 1.3	0.29
Thermoregulatory	1.3 ± 1.6	1.8 ± 2.3	0.18
Pupillomotor	0.5 ± 0.8	0.5 ± 0.8	0.32
Sexual function	0.8 ± 1.6	0.2 ± 0.9	0.002
SDQ score	5.6 ± 5.5	4.6 ± 4.4	0.24

The data are mean ± SD or n (%).

### 3.3 Drooling severity and patient perspectives on botulinum toxin therapy (Table 3)

Among droolers (*n* = 116), 62 were classified as having mild drooling and 54 as having severe drooling based on MDS-UPDRS Part II item 2 scores. The severe drooling group had significantly higher DSFS scores than the mild group (4.3 ± 1.7 vs. 2.4 ± 0.8, *p* < 0.001). Similarly, MDS-NMS item J-1 scores were significantly higher in the severe group (6.1 ± 4.1 vs. 1.0 ± 1.6, *p* < 0.001). MDS-NMS non-motor fluctuation severity was also higher in the severe group (1.2 ± 2.5 vs. 0.6 ± 1.2, *p* = 0.021). In contrast, MDS-UPDRS Part II item 3 and MDS-NMS item J-2 scores did not differ significantly between the groups. Regarding patient perceptions, 40 patients (34.5%) reported having problems with drooling. This proportion was significantly higher in the severe drooling group than in the mild group (57.4% vs. 14.5%, *p* < 0.001). Awareness of botulinum toxin therapy as a treatment option for drooling was low overall (14.7%) and did not differ significantly between the groups. However, willingness to receive botulinum toxin therapy was significantly higher in the severe drooling group compared with the mild group (35.2% vs. 9.7%, *p* < 0.001). Among patients who declined treatment (*n* = 91), the most common reason was that symptoms were not sufficiently bothersome to undergo injections (71.4%), followed by fear of injections (9.9%), limited knowledge about botulinum toxin therapy (6.6%), and other reasons (12.1%).

**Table 3:** Drooling-related clinical measures and patient perceptions of botulinum toxin therapy.

	Total patients		Mild drooling group⁎		Severe drooling group⁎⁎		*p* value
N	116		62		54		
Drooling Severity and Frequency Scale	3.3 ± 1.6		2.4 ± 0.8		4.3 ± 1.7		<0.001
MDS-UPDRS Part II item 3	0.4 ± 0.8		0.3 ± 0.7		0.6 ± 0.8		0.098
MDS-NMS item J-1	3.4 ± 4.0		1.0 ± 1.6		6.1 ± 4.1		<0.001
MDS-NMS item J-2	1.1 ± 2.0		1.0 ± 1.9		1.1 ± 2.2		0.81
MDS-NMS non-motor fluctuation severity	0.9 ± 1.4		0.6 ± 1.2		1.2 ± 2.5		0.021
Patient responses	Yes n (%)	No n (%)	Yes n (%)	No n (%)	Yes n (%)	No n (%)	
*I have problems with drooling.*	40 (34.5)	76 (65.5)	9 (14.5)	51 (85.5)	31 (57.4)	25 (42.6)	<0.001
*I am aware that botulinum toxin therapy is available as a treatment option for drooling.*	17 (14.7)	99 (85.3)	11 (17.7)	49 (82.3)	6 (11.1)	50 (88.9)	0.31
*I would be willing to receive botulinum toxin therapy for the treatment of drooling.*	25 (21.6)	91 (78.5)	6 (9.7)	54 (90.3)	19 (35.2)	37 (64.8)	<0.001
Reasons for declining botulinum toxin therapy of drooling (n = 91)	1) Not bothered enough to receive injections (n = 65; 71.4%) 2) Fear of injections (*n* = 9; 9.9%) 3) Limited knowledge of botulinum toxin therapy (*n* = 6; 6.6%) 4) Other (n = 11; 12.1%)						

The data are mean ± SD or n (%).

Abbreviations: NMS: non-motor symptom.

⁎ Patients with an MDS-UPDRS Part II item 2 score of 1–2 were defined as the mild drooling group.

⁎⁎ Patients with an MDS-UPDRS Part II item 2 score of 3–4 were defined as the severe drooling group.

**Table 4:** Clinical characteristics of droolers stratified by cluster analysis.

	Cluster A: Low SCOPA-AUT / Low SDQ	Cluster B: High SCOPA-AUT / High SDQ	Cluster C: High SCOPA-AUT / Low SDQ	*p* value
N (n = 116)	65 (56.0)	13 (11.2)	38 (32.8)	–
SCOPA-AUT total score	11.5 ± 3.6	24.9 ± 8.6^⁎⁎⁎^	23.1 ± 4.3^†††^	<0.001
SDQ score	4.7 ± 2.8	19.7 ± 4.6^⁎⁎⁎‡‡‡^	6.8 ± 3.1^†††^	<0.001
Male	42 (64.6)	8 (61.5)	24 (63.2)	0.97
Age, yrs	71.9 ± 9.2	71.8 ± 14.4	71.9 ± 9.7	0.81
Onset age, yrs	62.9 ± 10.7	62.2 ± 17.9	61.3 ± 11.3	0.54
Disease duration, yrs	9.0 ± 6.2	9.5 ± 5.1	10.6 ± 6.9	0.51
Treatment duration, yrs	6.5 ± 5.5	6.9 ± 4.7	7.7 ± 6.7	0.72
Hoehn & Yahr stage	2.7 ± 0.8	2.9 ± 0.6	3.2 ± 0.7^††^	0.006
LEDD	753.4 ± 481.9	771.7 ± 402.9	803.0 ± 516.9	0.57
Use of anticholinergic medication	6 (9.2)	2 (15.4)	1 (2.7)	0.26
History of diabetes	3 (4.6)	1 (7.7)	4 (10.5)	0.52
History of heart disease	5 (7.7)	2 (15.4)	4 (10.5)	0.66
MIBG myocardial scintigraphy				
H/M ratio early image (*n* = 99)	1.81 ± 0.54	2.20 ± 0.60	1.84 ± 0.48	0.056
H/M ratio delayed image (*n* = 95)	1.63 ± 0.58	2.08 ± 0.86	1.62 ± 0.60	0.10
Washout rate (*n* = 93)	54.0 ± 17.2	45.4 ± 16.9	56.7 ± 17.0	0.26
MDS-UPDRS Part II item 2	2.2 ± 0.9	2.9 ± 0.8	2.5 ± 1.0	0.033
MDS-UPDRS Part II item 3	0.3 ± 0.6	1.4 ± 1.2^⁎⁎⁎‡‡^	0.4 ± 0.7	<0.001
Drooling Severity and Frequency Scale	2.9 ± 1.4	4.1 ± 1.9	3.7 ± 1.7^†^	0.014
MDS-NMS item J-1	2.4 ± 3.0	5.0 ± 4.1^⁎⁎^	4.5 ± 4.9	0.009
MDS-NMS item J-2	0.7 ± 1.5	3.7 ± 3.3^⁎⁎⁎‡‡‡^	0.8 ± 1.5	<0.001
MDS-NMS non-motor fluctuation severity	0.8 ± 1.3	1.5 ± 1.6	0.9 ± 1.4	0.13

The data are mean ± SD or n (%).

^⁎⁎^*p* < 0.01, ^⁎⁎⁎^*p* < 0.001: A vs B. ^†^*p* < 0.0167, ^††^*p* < 0.01, ^†††^*p* < 0.001: A vs C. ^‡‡^*p* < 0.01, ^‡‡‡^*p* < 0.001: B vs C.

Above *p*-values were adjusted using the Bonferroni method.

### 3.4 Cluster-derived subgroups based on autonomic and dysphagia severity among droolers (Table 4 and Supplementary Fig. 1)

**Supplementary Fig. 1:**
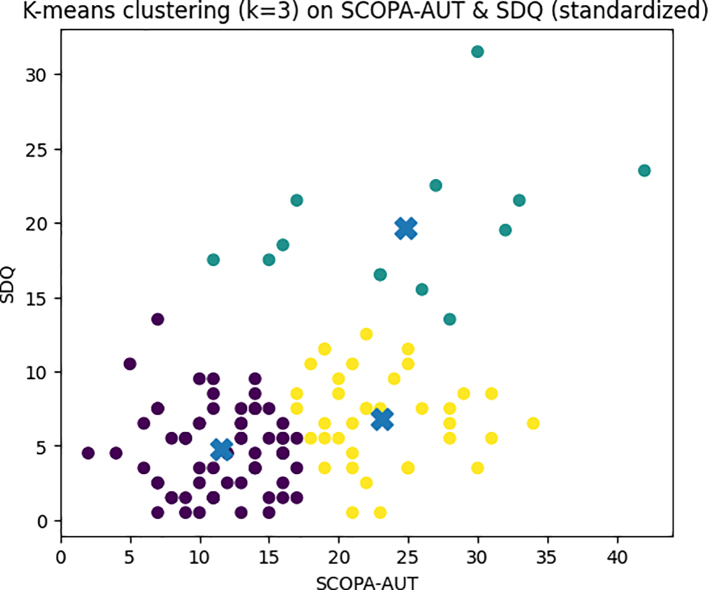
Clustering results based on the SCOPA-AUT total score and SDQ score.

The 116 PD patients with drooling were stratified into three clusters based on SCOPA-AUT and SDQ scores (Supplementary Fig. 1): cluster A (low SCOPA-AUT/low SDQ; *n* = 65, 56.0%), cluster B (high SCOPA-AUT/high SDQ; *n* = 13, 11.2%), and cluster C (high SCOPA-AUT/low SDQ; *n* = 38, 32.8%). As expected, autonomic symptom burden differed significantly across clusters, with SCOPA-AUT total scores higher in clusters B and C than in cluster A (both Bonferroni-adjusted *p* < 0.001). SDQ scores were highest in cluster B and significantly greater than those in clusters A and C (both adjusted *p* < 0.001). Demographic variables (sex, age, age at onset), disease duration, treatment duration, LEDD, anticholinergic medication use, and medical comorbidities did not differ significantly among clusters. HY stage was higher in cluster C than in cluster A (adjusted *p* < 0.01). MDS-UPDRS Part II scores differed among clusters, with a marked increase in item 3 scores in cluster B compared with cluster A (adjusted *p* < 0.001). For MDS-UPDRS Part II item 2, a significant overall difference was observed between clusters B and A (*p* = 0.033, Cohen's d = 0.74); however, no pairwise comparisons remained significant after Bonferroni correction. Drooling severity also varied across clusters: DSFS scores were higher in cluster B than in cluster A (*p* = 0.014) and tended to be higher in cluster C than in cluster A, although this difference did not reach statistical significance after correction. Non-motor symptom measures were significantly elevated in cluster B. MDS-NMS item J-1 scores were higher in cluster B than in cluster A (*p* < 0.01), and item J-2 scores were higher in cluster B than in both clusters A and C (both adjusted *p* < 0.001). By contrast, non-motor fluctuation scores did not differ significantly among clusters. MIBG myocardial scintigraphy indices showed a trend toward higher early H/M ratios in cluster B (*p* = 0.056, Cohen's d = 0.68), whereas delayed H/M ratios and washout rates did not differ significantly across clusters.

## 4 Discussion

In this questionnaire-based study of Japanese outpatients with PD, chronic sialorrhea was common and was associated with male sex, longer disease and treatment duration, greater motor severity, and a substantially higher burden of autonomic symptoms. Importantly, the association between drooling and autonomic dysfunction remained significant even after exclusion of the drooling-related SCOPA-AUT item, indicating that this relationship reflects a broader autonomic phenotype rather than simple symptom item overlap. Droolers also reported a significantly higher dysphagia burden. Despite these associations, only approximately one-third of patients classified as droolers perceived drooling as problematic, and awareness of botulinum toxin therapy was limited. Furthermore, an unsupervised clustering approach stratified droolers into three clinically interpretable subgroups based on autonomic symptom burden and dysphagia severity, highlighting heterogeneity in the mechanisms and experiences underlying drooling in PD.

### 4.1 Sialorrhea in PD and the role of impaired clearance

The present findings are consistent with the prevailing concept that sialorrhea in PD primarily reflects impaired saliva clearance rather than hypersalivation. [13], [14] Droolers exhibited higher SDQ scores, supporting an association with swallowing dysfunction. However, the cluster-based analysis indicates that dysphagia alone does not fully account for drooling. In particular, we identified a subgroup characterized by low autonomic symptom burden and low dysphagia burden (cluster A) and another subgroup characterized by high autonomic symptom burden with relatively low dysphagia burden (cluster C). These findings suggest that partially independent mechanisms may lead to clinically relevant drooling. Possible contributors include reduced spontaneous swallowing frequency, oromotor bradykinesia or rigidity affecting lip seal and bolus handling, and posture-related pooling. [15] The higher Hoehn & Yahr stage observed in cluster C further supports the contribution of motor severity to impaired oral-phase clearance, even in the absence of prominent subjective dysphagia.

Taken together, these findings indicate that dysphagia alone does not fully explain the occurrence of drooling in PD. Rather, multiple mechanisms—including impaired swallowing, autonomic dysfunction, and motor-related oral-phase impairment—may contribute to reduced saliva clearance. This supports the concept that drooling represents a heterogeneous clinical phenomenon rather than a single pathophysiological entity.

### 4.2 Autonomic dysfunction as a key correlate of drooling

A notable finding of this study was the strong association between drooling and overall autonomic symptom severity. Autonomic symptom burden assessed by the SCOPA-AUT remained significantly associated with drooling even after exclusion of the drooling-related item, indicating that the observed relationship reflects broader autonomic involvement rather than simple overlap in symptom assessment. While drooling in PD has traditionally been interpreted as a consequence of impaired saliva clearance due to oropharyngeal motor dysfunction, our findings suggest that autonomic dysfunction may also contribute to its pathophysiology. In addition to dysphagia severity, gastrointestinal and urinary autonomic domains were independently associated with drooling, suggesting that drooling may reflect more widespread non-motor involvement. Previous studies, including the recent report by Igami et al., [17] have suggested that drooling reflects overall disease severity in PD. In contrast, our results indicate that autonomic dysfunction remained independently associated with drooling even after adjustment for disease severity, suggesting that drooling may represent a specific non-motor phenotype rather than simply a marker of advanced disease. Importantly, autonomic dysfunction in this context does not necessarily imply increased salivary secretion. Rather, it may reflect broader non-motor/autonomic involvement that indirectly exacerbates impaired saliva clearance through mechanisms such as gastrointestinal dysmotility, orthostatic symptoms leading to reduced activity or postural changes, and sleep-related factors that diminish effective swallowing. Droolers also exhibited lower MIBG H/M ratios on both early and delayed images, consistent with more severe cardiac sympathetic denervation and may reflect broader Lewy body pathology, its relationship with specific non-motor symptoms remains incompletely understood. Previous studies have reported correlations between MIBG findings and SCOPA-AUT–derived autonomic symptoms. [24] Our results therefore raise the possibility that drooling may be associated with more widespread autonomic neuropathology. Notably, MIBG indices did not significantly differ among cluster-defined drooler subgroups, although a non-significant trend toward higher early H/M ratios was observed in the high SCOPA-AUT/high SDQ cluster. This discrepancy may reflect limited statistical power within small clusters, variability in the timing of imaging relative to symptom assessment, and heterogeneity in the mechanisms linking cardiac sympathetic denervation to specific autonomic symptom profiles. Future studies incorporating standardized imaging schedules and comprehensive autonomic testing are warranted.

### 4.3 Sex differences in drooling and autonomic burden

Droolers were more often male, and male patients exhibited greater drooling-related disability and higher autonomic symptom burden overall. These findings are consistent with previous reports indicating a higher prevalence of clinically significant sialorrhea among males with PD. [15], [17] Potential explanations include sex-related differences in autonomic involvement, posture or oromotor characteristics, or differences in symptom reporting thresholds. [25], [26], [27] In our cohort, males demonstrated lower MIBG H/M ratios and a higher prevalence of heart disease, suggesting greater cardiac sympathetic denervation and cardiovascular vulnerability. However, the literature on sex differences in autonomic dysfunction in PD remains inconsistent; for example, Meksi et al. reported more pronounced autonomic impairment in females. [28] Although causal inference is not possible, our findings support considering sex as a clinically relevant factor when screening for drooling and discussing management options.

### 4.4 Patient perception and the treatment gap in the era of botulinum toxin therapy

A clinically actionable observation from this study is the apparent gap between scale-based symptom classification and patient-perceived burden. Only approximately one-third of droolers self-identified drooling as problematic, and awareness of botulinum toxin therapy was very limited. Several factors may contribute to this treatment gap. First, the operational definition of drooling in this study included patients with mild symptoms that may not be perceived as clinically significant. Second, patients with PD often prioritize other disabling motor or non-motor symptoms, potentially leading to under-recognition or underreporting of drooling. In addition, drooling is a socially sensitive symptom that patients may hesitate to discuss unless specifically asked. Importantly, willingness to receive botulinum toxin therapy was significantly higher among patients with more severe drooling, indicating that perceived symptom burden strongly influences treatment acceptance. Given that botulinum toxin therapy is an effective and guideline-supported treatment for chronic sialorrhea in PD, these findings highlight the importance of proactive screening and patient education in routine clinical practice. Simple screening questions and clear explanations regarding the safety and benefits of botulinum toxin therapy may help bridge the gap between symptom severity, patient perception, and treatment uptake.

### 4.5 Clinical implications of cluster-derived phenotypes

The cluster-derived phenotypes offer a pragmatic framework for stratifying droolers in PD: (A) low autonomic/low dysphagia burden, (B) high autonomic/high dysphagia burden, and (C) high autonomic/low dysphagia burden. Cluster B exhibited the greatest overall drooling-related disability and non-motor symptom burden, suggesting that these patients may benefit from multidomain intervention, including botulinum toxin therapy, targeted dysphagia assessment and rehabilitation, and structured management of autonomic symptoms. In contrast, cluster C may require focused evaluation of motor and oral-phase contributors, posture, and autonomic screening rather than dysphagia-centered intervention alone. Such phenotyping may help clinicians tailor assessment and counseling rather than applying a uniform approach to all patients with drooling.

### 4.6 Limitations

This study has several limitations. First, the drooler/non-drooler classification was defined using a single self-report item (MDS-UPDRS Part II item 2), which may be influenced by patient awareness and reporting style, and objective measures of drooling were not available for all patients. Second, dysphagia was assessed using a questionnaire (SDQ) rather than instrumental swallowing evaluations, such as videofluoroscopic swallowing study or fiberoptic endoscopic evaluation of swallowing, which were not systematically performed. Third, the cross-sectional, single-center design limits causal inference and generalizability. Fourth, the timing of MIBG scintigraphy relative to symptom assessment varied, and imaging was not available for all participants. Fifth, the cluster analysis was based on two variables with a pre-specified number of clusters; although the resulting phenotypes were clinically interpretable, external validation and sensitivity analyses using alternative approaches are needed. In particular, the number of patients in cluster B was relatively small (*n* = 13), which may limit the stability, robustness, and reproducibility of the cluster-derived phenotypes.

Finally, the modest sample size within some clusters may have limited statistical power.

### 4.7 Conclusions

Chronic sialorrhea in PD is associated with male sex, greater disease severity, higher dysphagia burden, and importantly more severe autonomic symptoms, supporting the concept that drooling in PD reflects a complex interplay between impaired clearance and broader non-motor/autonomic dysfunction. Patient awareness of drooling and knowledge of botulinum toxin therapy were limited despite the availability of an approved treatment, highlighting an unmet need for systematic screening and patient education. Cluster-derived phenotypes based on autonomic and dysphagia burden may facilitate more personalized assessment and management strategies for drooling in PD.

The following are the supplementary data related to this article.

Supplementary material 2

## Author Contribution

MS had full access to all of the data in the study and takes responsibility for the integrity of the data and the accuracy of the data analyses. Concept and design: SO and MS. Acquisition, analysis, or interpretation of data: SO and MS. Drafting of the manuscript: SO and MS. Statistical analysis: SO. Administrative, technical, or material support: SO, TN, YN, and MS. Supervision: YN, JN, and MS. Critical revision of the manuscript for important intellectual content: All authors.

## Funding/Support

This research did not receive any specific grant from funding agencies in the public, commercial, or not-for-profit sectors.

## Role of the Funder/Sponsor

None.

## Ethical Compliance Statement

Ethical approval for this study was obtained from the institutional ethics committee of Keio University School of Medicine (approval number # 20231166). The requirement for written informed consent was waived owing to the retrospective nature of the analysis, and information about the study was disclosed to all patients, who were given the opportunity to opt out. We confirm that we have read the Journal's position on issues involved in ethical publication and affirm that this work is consistent with those guidelines.

## Author Declaration

Parkinsonism & Related Disorders is committed to proper scientific conduct and the protection of animal and human research subjects. Submission of this manuscript implies compliance with the following ethical requirements. Please affirm that you are representing all of the authors in stating compliance with these policies by checking the box at the end of this section.

1. Studies with human subjects must have been conducted in accordance with the Declaration of Helsinki. All persons must have provided informed consent prior to being included in the study.

2. Studies with animal subjects must have been conducted in accordance with the Guide for the Care and Use of Laboratory Subjects as adopted by the US National Institutes of Health and/or according to the requirements of all applicable local, national and international standards.

3. Protocols with animal or human subjects must have been approved by the relevant local committee(s) charged with ensuring subject protection. Studies that entail pain or distress will be assessed in terms of the balance between the distress inflicted and the likelihood of benefit.

4. The authors declare that the manuscript is original, that it is not being considered for publication elsewhere, and that it will not be submitted elsewhere while still under consideration for Parkinsonism & Related Disorders or after it has been accepted by Parkinsonism & Related Disorders.

5. All authors have seen and approved the manuscript in the form submitted to the journal. The authors declare that they have conformed to the highest standards of ethical conduct in the submission of accurate data and that they acknowledge the work of others when applicable.

6. All sources of financial support for the work have been declared in the Acknowledgements section of the manuscript. Any additional conflicts of interest must also be declared. Please include declarations of any consultancy or research funding received from relevant companies from three years prior to performance of the research until the time of manuscript submission. If the research is supported by internal funds, that should be stated as well.

To indicate compliance with the preceding declaration and that you have obtained agreement from all of the authors of this paper to declare their compliance as well, please place an x here: **X**

In cases of uncertainty please contact an editor for advice.

## Declaration of competing interest

The authors declare that they have no known competing financial interests or personal relationships that could have appeared to influence the work reported in this paper.

## Data Availability

The datasets generated and/or analyzed during the current study are available from the corresponding author upon reasonable request.
