# Supplementary material for: Clinical and autonomic correlates of chronic Sialorrhea in Parkinson's disease: a questionnaire-based study with cluster analysis of autonomic and dysphagia burden
Source: Clin Park Relat Disord. 2026 Jun 18;15:100467. doi: 10.1016/j.prdoa.2026.100467 (PMC13320329; doi:10.1016/j.prdoa.2026.100467)
Supplement: Supplementary material 2 [file mmc2.docx]

**Suppl. Table S1.** Multivariable logistic regression analysis of factors associated with drooling: Model 1 including SCOPA-AUT total score, SDQ score, and H/M ratio, and Model 2 including SCOPA-AUT subdomain scores

|  | Model 1 OR (95% CI) | *p* value | Model 2 OR (95% CI) | *p* value |
| --- | --- | --- | --- | --- |
| SCOPA-AUT total score excluding the drooling item | 1.04 (1.00–1.08) | 0.03 | - | - |
| SDQ score | 1.33 (1.16–1.52) | <0.001 | - | - |
| H/M ratio delayed image | 0.75 (0.39–1.46) | 0.40 | - | - |
| SCOPA-AUT subdomain scores |  |  |  |  |
| Gastrointestinal excluding the drooling item | - | - | 1.21 (1.06–1.37) | 0.004 |
| Urinary | - | - | 1.11 (1.00–1.24) | 0.049 |
| Cardiovascular | - | - | 1.17 (0.87–1.57) | 0.30 |
| Thermoregulatory | - | - | 0.99 (0.82–1.20) | 0.90 |
| Pupillomotor | - | - | 0.72 (0.46–1.13) | 0.16 |
| Sexual function | - | - | 1.02 (0.79–1.32) | 0.88 |
| Age | 1.04 (0.99–1.08) | 0.12 | 1.03 (0.99–1.07) | 0.17 |
| Sex | 0.57 (0.25–1.27) | 0.17 | 0.56 (0.28–1.12) | 0.10 |
| Disease duration | 1.00 (0.92–1.09) | 0.98 | 1.02 (0.96–1.09) | 0.47 |
| Hoehn & Yahr stage | 0.93 (0.55–1.59) | 0.79 | 1.22 (0.78–1.93) | 0.39 |
| LEDD (per 100 mg increase) | 1.06 (0.96–1.18) | 0.25 | 1.06 (0.97–1.15) | 0.19 |

Abbreviations: CI: confidence interval; H/M: heart-to-mediastinum; LEDD: levodopa equivalent daily dose; OR: odds ratio; SDQ: Swallowing Disturbances Questionnaire; SCOPA-AUT: Scales for Outcomes in Parkinson’s Disease–Autonomic.

All models were adjusted for age, sex, disease duration, Hoehn and Yahr stage, and LEDD.
